# Development and Validation of a Questionnaire to Assess Secondary Food Habits Associated With Smoking

**DOI:** 10.7759/cureus.112500

**Published:** 2026-07-11

**Authors:** Manoj Prabhakar, Kavitha B, Preetha Elizabeth Chaly, Ramesh Kumar SG, Ardhanaari M

**Affiliations:** 1 Department of Oral Pathology and Microbiology, Meenakshi Ammal Dental College and Hospital, Meenakshi Academy of Higher Education and Research, Chennai, IND; 2 Department of Public Health Dentistry, Meenakshi Ammal Dental College and Hospital, Meenakshi Academy of Higher Education and Research, Chennai, IND; 3 Department of Public Health Dentistry, Tamil Nadu Government Dental College and Hospital, Chennai, IND; 4 Department of Psychiatry, Meenakshi Medical College Hospital and Research Institute, Meenakshi Academy of Higher Education and Research, Kanchipuram, IND

**Keywords:** dietary habits, eating behavior, food habits, questionnaire, smoking

## Abstract

Background: Food intake patterns among smokers represent a complex behavioral phenomenon, wherein, beyond general dietary habits, smokers often engage in specific food consumption during or immediately after smoking episodes. These “secondary food substances” include sugary products such as chocolates, candies, sweeteners, and chewing gum, menthol-based items, hot and cold beverages, carbonated drinks, mouth fresheners, and occasionally smokeless tobacco products. Such practices may be driven by factors including the desire to mask the odor or taste of smoke, sensory gratification, cravings, accessibility, peer influence, and media exposure.

Aim: This study aimed to develop a valid and reliable questionnaire for assessing secondary food consumption behavior during or immediately after smoking.

Methods: A systematic approach was adopted to design a structured questionnaire that focused on secondary food habits associated with smoking episodes. Item generation was based on a literature review and behavioral constructs related to secondary food substance use. The construct includes five categories: types of secondary food substances; frequency of use; reasons for using such food substances; attempts to quit; and craving, tolerance, and dependence developed toward the food substances. The entire set of items was refined and validated through focus group discussions, content validity, construct validity, internal consistency, and test-retest reliability.

Results: The questionnaire comprised 10 items and demonstrated excellent content validity, with an overall Content Validity Index of 0.97. All items exhibited factor loadings above 0.50, confirming satisfactory construct validity. Itemwise correlation coefficients ranged from 0.11 to 0.81, indicating acceptable internal consistency. Furthermore, the intraclass correlation coefficient values ranged from 0.72 to 0.91, reflecting high test-retest reliability of the instrument.

Conclusion: Secondary food consumption habits associated with smoking may contribute to additional dependencies and adversely affect nutritional health, thereby compounding the harmful effects of the primary habit, smoking. The present study addresses a critical gap in the literature by introducing a dedicated assessment tool to systematically evaluate these behaviors, with potential applications in research and behavioral interventions.

## Introduction

Smoking and its association with eating and dietary behavior is a complex, multifactorial relationship. Smokers tend to exhibit varied dietary patterns, some showing extensive eating while others with loss of appetite. Nicotine, a key component in tobacco, suppresses appetite and affects food preferences, often triggering cravings for high-fat food, sugary items, processed food, and complex carbohydrates [1,2]. The combination of tobacco use and improper eating behavior is a major health concern, as their combined effects can decrease life expectancy. In addition to the independent risks related to cigarettes, smoking is also highly associated with obesity-related behaviors, including an unhealthy diet. An emerging body of evidence has demonstrated cross-substance craving for highly palatable foods among smokers [2]. It is to be noted that some of the smokers tend to consume specific foods or beverages during or immediately after smoking due to varying reasons. This kind of unhealthy eating behavior that happens secondarily, during or immediately after the primary habit (smoking), may be termed "secondary food habits."

Secondary food habits, which are anecdotal findings, include any snack item, hot or cold drinks, carbonated beverages, sugary items such as chocolates, candies, sweeteners, and chewing gums, menthol-based products, and mouth fresheners, which smokers usually consume during or immediately after the smoking episode. Preference to take these food substances by an individual who smokes can be due to various reasons, such as the desire to mask the smell or taste of smoke, specific food cravings, peer influence, easy availability, and accessibility of food substances. Socioeconomic status of the individual/family, level of education, and cultural characteristics also influence the choice of food substances. Further, food consumption disparities and the type of food choice also vary based on the country of origin, and urban or rural setup of living [3]. Both smoking and the associated eating behavior have their own effect on oral health and may lead to several oral diseases. As a matter of fact, incidentally, these two risk factors are associated with one another, and the interconnection may be understood through the common risk factor approach [4]. In addition to the primary habit (smoking), some of the smokers get addicted to these secondary food habits over a period of time, which may eventually lead to unhealthy eating behavior. This secondary eating behavior pattern among the smokers is so far unexplored and remains a silent epidemic.

Although there are different food addiction scales developed to assess the eating behavior of an individual, addiction to a specific type of food during or immediately after smoking has not been measured or documented in the literature. Hence, there is a need for the development of a unique questionnaire that measures the "secondary food habit" usage among smokers. Development of a validated questionnaire, as in this case, will provide accurate, reliable, and comparable data to derive strong conclusions and recommendations to the general public, counselors, and policy makers. The present paper is thus aimed at developing and validating a questionnaire to assess the prevalence, type, and usage pattern of secondary food substances, as well as the dependency or addictive-like behavior of the individuals developed toward such food substances that are used during or immediately after smoking.

## Materials and methods

Study design

This cross-sectional study was conducted among smokers in Chennai City. The study involved the development of a questionnaire through the formation of a conceptual framework, the development of an item pool, the refinement of the items, focus group discussion, pretesting, validation, and reliability. The study protocol was submitted to the Institutional Ethical Committee, and ethical clearance was obtained after answering the queries raised by the panel members (Ref. No.: MADC/IEC/I/07/2025). All participants involved in the study provided informed consent.

Study duration

The study was conducted over 10 months, from May 2025 to February 2026. The initial two months were dedicated to preliminary discussions, development of the conceptual framework, and identification of key constructs. The subsequent three months involved the development and refinement of the item pool through literature review, expert consultations, focus group discussions, and pretesting. Pilot testing of the questionnaire was carried out over the next three months to assess the feasibility, clarity, and comprehensibility of the items. The final two months were utilized for validity and reliability testing, data collection, and statistical analysis.

Sampling methodology and eligibility criteria

The participants with the habit of smoking were purposively selected from different field settings like refreshment outlets, educational institutions, software industries, and factories. The participants who were above 18 years and had at least one year of smoking habit were included in the study. Those participants who had the habit of both smoking and smokeless tobacco were excluded. The need and purpose of the study were explained to the smokers who met the eligibility criteria, and a subject information sheet was provided. Only those participants who signed the informed consent form were included in the study.

Sample size

Based on the sample size suggested by Terwee et al. [5], the number of samples for questionnaire validation studies must be seven times the number of items generated. Since the questionnaire had 11 items, 77 samples were sufficient to perform construct validity analysis. However, about 172 participants were approached to achieve the sample size, and among them, about 133 reported having secondary food habits. Hence, the responses from these 133 samples were analyzed to assess construct validity and reliability.

Formation of a conceptual framework

Key components involved in the study were identified as core constructs, and the relationship between smoking and secondary food habits was explored through the conceptual framework. Specific variables included in the framework were appropriately matched with the research question. A set of item pool comprising related questions was generated and included under each construct.

Development of the item pool

A complete, comprehensive literature search was conducted by the principal investigator to identify secondary food habits associated with smoking. Based on the results obtained, the questionnaire items were generated. A panel team consisting of a Preventive and Public Health Dentist, Tobacco Cessation and Counseling personnel, an Oral Physician and Pathologist, a Psychiatrist, and a Statistician was present throughout the item-generation process.

A preliminary form of questions related to secondary food habits was developed, focusing on the type, duration, and frequency of the habit. Some of the questions were related to craving, withdrawal, dependency, and tolerance toward the habit. These items were modified and adopted from the previous scales to match the construct of the present study. The item pool included a mix of questions: full-sentence items that were open-ended or closed-ended to elicit the most appropriate and comprehensive responses from participants. A total of 15 items were included in the preliminary version of the tool.

Refinement of the item pool

Questions generated at the preliminary stage for each construct were refined based on feedback and responses from the focus group discussion and pretesting. Additional items were introduced, and existing items were revised, resulting in a finalized questionnaire comprising 10 items. The responses for the items were recorded as open-ended for one question and on a three-point Likert scale for the rest of the items. The items under each domain, along with their modifications, are given in Table 1.

**Table 1:** Items involved in the Secondary Food Substances questionnaire

Constructs/domains	Initially considered items	Newly added items	Modified/removed items	Final items in the questionnaire
Domain 1: type of secondary food substances	Type of secondary food substance used along with smoking. Any change in the type of secondary food substance used during smoking	Reason for changing the secondary food substance	Repeated questions in the context of changing the secondary food substances were removed. The term “product” was replaced with “secondary food substances” in all respective items	Item 1: What food substance(s)/beverage(s) do you use along with/immediately after smoking? Item 5: Have you changed using the food substance/beverage any time during the course of smoking?
Domain 2: frequency of usage	Regular or irregular use of secondary food substances after every smoking episode. Initiation of secondary food substance use (from the beginning or during the course of a smoking habit)	-	If not used regularly, how frequently per day during or after every smoking episode	Item 2: Do you use this food substance/beverage regularly after every smoke? Item 3: When did you start using this kind of food substance/beverage along with/immediately after smoking?
Domain 3: reasons	Masking, peer influence, craving, and easy availability of the food substances were considered as options for indulging in secondary food habit	Media influence was included as another option	-	Item 4: What makes you to indulge in this habit during/soon after smoking?
Domain 4: quit attempts	Attempt taken to quit the use of secondary food substances. Reduction in secondary food substance use	-	-	Item 6: Have you ever attempted to quit the use of a food substance/beverage which you consume along with/immediately after smoking? Item 7: Have you tried to cut down or reduce having certain food substance/beverages which you consume along with/immediately after smoking?
Domain 5: craving, tolerance, and dependency	Feelings of low mood or depression associated with not using secondary food substances. Increase in the amount or frequency of secondary food substance use. Strong urge to consume specific food substances	The level of stress was included as a separate item	Likert scale with the responses “Strongly Disagree” to “Strongly Agree” was used initially to record craving, tolerance, and dependency. Later, it was replaced with Yes or No options. Likert scale from 1 to 5 was used initially to record stress level. Later, the Depression, Anxiety, and Stress Scale [6] was used	Item 8: Do you feel low or depressed if you don’t take this food substance/beverage along with/immediately after smoking? Item 9: Have you increased the dose or frequency of the food substance/beverage that you consume along with/immediately after smoking? Item 10: Have you felt or experienced a strong urge to consume food substance/beverage along with/immediately after smoking that you could not think of anything else?

Focus Group Discussion

A specific group of participants with secondary food habits associated with smoking was selected, and a discussion was conducted to identify any difficulty in understanding the questions in terms of clarity and relevance. The group consisted of 12 participants, along with a public health dentist, a psychiatrist, and a statistician for their expert opinions. Any new responses or suggestions from the members were added as new items to the main item pool. The group discussion was audiovisually recorded for further reference.

Pretesting

A small group of participants (n = 20) was administered the questionnaire to assess whether all items were easy to understand and to evaluate their ability to accurately interpret the meanings of the words and any technical terms used. Any questions that lacked clarity and those the participants felt were least important were either revised or removed accordingly. Participants who were involved in the habit of smoking for at least one year, along with associated secondary food habits, were involved in the pretesting phase. The questions were administered to the participants in a few common places, as well as in the outpatient department of the private dental institution.

Validity analysis

Content Validity

The developed questionnaire underwent both qualitative and quantitative content validation. Expert members from different specialties reviewed the questionnaire on the basis of clarity and comprehension, and checked for grammatical errors. After qualitatively refining the questionnaire based on members' suggestions, the final draft was prepared and resubmitted to the panel for review. Each item’s relevancy and appropriateness were checked by the panel members. Based on the responses, Item-Wise Content Validity Index Score and Scale-Wise Content Validity Index Score were calculated. The questionnaire was further revised until a Content Validity Index (CVI) of more than 0.80 was obtained [7].

Construct Validity

Based on the theoretical framework, the questionnaire was designed with five domains, and a confirmatory factor analysis was conducted to validate this underlying construct structure. An item was considered part of the domain when a factor load of ≥0.5 was obtained.

Reliability analysis

Internal Consistency

The internal consistency was measured using Cronbach’s alpha and interitem correlation. The recommended range for interitem correlation was reported as 0.2-0.5.

Test-Retest Reliability

A total of 30 smokers who had the habit of secondary food habits associated with smoking were randomly selected to assess test-retest reliability. The same questionnaire was administered to the 30 selected smokers after two weeks, and their responses were recorded. The correlation of scores obtained at two time points was assessed using the intraclass correlation coefficient (ICC).

Statistical analysis

Data entry was performed in a Microsoft Excel spreadsheet (Microsoft Corporation, Redmond, WA), and analysis was performed using the Statistical Package for the Social Sciences, version 27.0 (IBM Corp., Armonk, NY). The categorical variables were expressed as frequencies (n) and percentages (%), and continuous variables as means and standard deviations. The internal consistency of the items was assessed using the Spearman correlation matrix, and ICC was used to assess test-retest reliability. A p value of <0.05 was considered statistically significant.

## Results

Sociodemographic details

Table 1 shows the sociodemographic details of study participants. The mean age of the study participants was 37.33 + 12.49. About 168 (97.7%) of them were male participants, and only about four (2.3%) of them were female participants in the present study. About 119 (69.2%) were married, and 53 (30.8%) were single. The median monthly income of the study participants was 45,000 Indian Rupees. Among the 172 participants included, about 133 (77.3%) had secondary habits (Table 2).

**Table 2:** Sociodemographic details of study participants SD: standard deviation; IQR: interquartile range

Parameter	Value
Age, mean + SD	37.33 + 12.49
Gender, n (%)	
Male	168 (97.7)
Female	4 (2.3)
Education	
Below secondary education	20 (11.6)
Secondary and higher secondary education	25 (14.5)
Undergraduate	108 (62.8)
Postgraduate	19 (11)
Marital status, n (%)	
Married	119 (69.2)
Single	53 (30.8)
Monthly income (in Indian National Rupees), mean + SD; median (IQR)	71,663.53 + 80,705.06; 45,000 (3,000-8,000)
Prevalence of secondary food habits	133 (77.3)

Validity assessment

Content Validity Analysis

 The CVI scores of each item ranged from 0.84 to 1.0 across all items, indicating good content validity. The questionnaire’s overall CVI score was 0.97 (Table 3).

**Table 3:** CVI scores of the items in questionnaire CVI: Content Validity Index; Q-CVI: Question/Overall Content Validity Index; I-CVI: Item-Level Content Validity Index

Items	I-CVI score
Item-1	1.0
Item-2	0.84
Item-3	1.0
Item-4	0.90
Item-5	1.0
Item-6	1.0
Item-7	1.0
Item-8	1.0
Item-9	1.0
Item-10	1.0
Overall questionnaire (Q-CVI score)	0.97

Construct Validity Analysis

**Figure 1:**
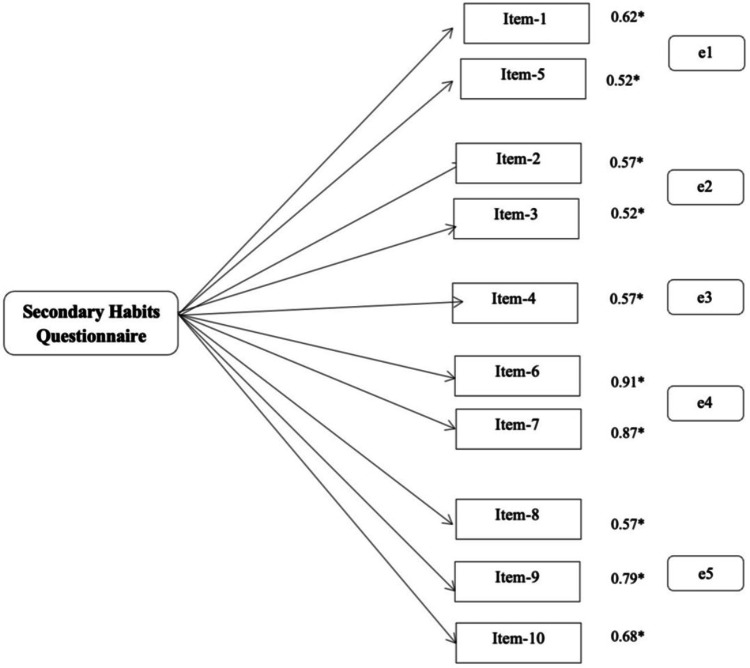
Confirmatory factor analysis of the questionnaire (five factor model for the items in the questionnaire) ^*^The factor loading values

Construct validity assessment was done among 133 samples who admitted the presence of secondary food habits associated with smoking. Figure 1 shows the five-factor model of the questionnaire items, along with their respective factor loadings. Domain 1, which focuses on the types of secondary food substances, had two items (Items 1 and 5). Domain 2, which assesses the frequency of use of secondary food substances, had two items (Items 2 and 3). Domain 3, which records the reason for using the secondary food substances, had only one item (Item 4). Attempts to quit secondary food habits were included in Domain 4, which had two items (Items 6 and 7). Domain 5, which recorded craving, tolerance, and dependence on secondary food habits, had three items (Items 8-10) (Figure 1).

Reliability assessment

Internal Consistency

The internal consistency of the questionnaire is represented in Table 4. Item 1 was an open-ended question and was therefore not included in the internal consistency assessment. The correlation coefficient of all items ranged from 0.11 to 0.81. There was a strong, significant correlation between Items 6 and 7 (correlation coefficient = 0.81; p < 0.001). A strong correlation was also observed between Items 8 and 9 (correlation coefficient = 0.81; p < 0.001) (Table 4).

**Table 4:** Internal consistency for the items in the questionnaire ^*^Correlation coefficient is statistically significant

Item	Item-2	Item-3	Item-4	Item-5	Item-6	Item-7	Item-8	Item-9	Item-10
Item-2	1.0	-	-	-	-	-	-	-	-
Item-3	0.54^*^	1.0	-	-	-	-	-	-	-
Item-4	0.67^*^	0.77^*^	1.0	-	-	-	-	-	-
Item-5	0.45^*^	0.13	0.47^*^	1.0	-	-	-	-	-
Item-6	0.71^*^	0.21	0.21	0.65^*^	1.0	-	-	-	-
Item-7	0.21	0.62^*^	0.13	0.32^*^	0.81^*^	1.0	-	-	-
Item-8	0.12	0.22	0.11	0.17	0.61^*^	0.44^*^	1.0	-	-
Item-9	0.61^*^	0.12	0.45	0.57^*^	0.61^*^	0.45^*^	0.81^*^	1.0	-
Item-10	0.24	0.21	0.35	0.37	0.22	0.21	0.65^*^	0.44^*^	1.0

Test-Retest Reliability

Table 5 shows the test-retest reliability of the questionnaire along with ICC values. The ICC values for the items in the questionnaire ranged from 0.72 to 0.91. This indicates high reliability of the questionnaire during tests and retests.

**Table 5:** Test-retest reliability of the questionnaire ^*^p value of <0.05 is considered statistically significant

Items	Intraclass correlation coefficient	p value
Item-1	0.76	0.02^*^
Item-2	0.72	0.03^*^
Item-3	0.91	<0.001^*^
Item-4	0.84	<0.001^*^
Item-5	0.90	<0.001^*^
Item-6	0.85	<0.001^*^
Item-7	0.82	<0.001^*^
Item-8	0.80	<0.001^*^
Item-9	0.81	0.01^*^
Item-10	0.77	0.01^*^

## Discussion

Eating behavior among smokers is heterogeneous and multifactorial, with a subset of individuals demonstrating a consistent pattern of food or beverage consumption during or immediately following smoking episodes. Engagement in such practices may be driven by factors including the desire to mask residual odor, modulate the sensory experience of smoke, or enhance overall satisfaction. In certain cases, these behaviors may evolve into conditioned preferences or dependencies, wherein individuals report smoking as incomplete in the absence of specific accompanying substances. Commonly reported items include hot or cold beverages, highly processed foods, sweeteners, chewing gums, and menthol-containing products. Beyond the established deleterious effects of smoking itself, the habitual consumption of these adjunct food substances may confer additional health risks, thereby warranting further investigation.

Smoking exerts a significant influence on food choices through a combination of physiological, neurochemical, and psychological mechanisms, largely mediated by nicotine. These processes contribute to the distinct dietary preferences and intake patterns observed among smokers. The tendency to consume specific foods or beverages during or immediately after smoking may be primarily linked to dopamine, a key neurotransmitter involved in reward, motivation, and behavioral reinforcement. Both smoking and the intake of sugary substances activate dopaminergic pathways, enhancing the perception of pleasure. Following smoking, nicotine-induced dopamine release sensitizes the brain’s reward system, potentially intensifying the sensory appeal of sweet or carbonated beverages. This interaction may facilitate the development of a reinforced behavioral loop associated with reward-seeking [8,9]. In addition, smoking has been associated with alterations in taste perception and craving for specific types of food. Evidence suggests that cigarette smoking may impair gustatory sensitivity and that smokers may exhibit reduced sensitivity to sweet and bitter tastes, which could promote increased consumption of highly palatable and energy-dense foods during smoking episodes [2]. Furthermore, psychological factors such as stress and anxiety, along with socioenvironmental influences including peer-related dietary practices, may also play a role in shaping food consumption behaviors among smokers [10,11].

Food choice is complex, and various biological, emotional, cognitive, cultural, and social factors contribute to the determination of a given nutritional style [12]. Addiction to a particular type of food is based on the addictive potential of certain food ingredients, as well as the addictive-like responses caused by highly processed foods. Various questionnaires were used to assess food addiction and its related behaviors, such as the Addiction-like Eating Behavior Scale, Grazing Questionnaire, Repetitive Eating Questionnaire, and Short Inventory of Grazing [13]. The most commonly used specific measure to assess food addiction is the Yale Food Addiction Scale (YFAS), which was developed by Schulte and Gearhardt [14] and which adapted the Diagnostic and Statistical Manual of Mental Disorders, Fourth Edition Text Revision (American Psychiatric Association, 2000) criteria for substance use disorders. Later, a modified version of YFAS 2.0 was developed with a maximum of 11 items, reflecting the Diagnostic and Statistical Manual of Mental Disorders, Fifth Edition, diagnostic criteria [14,15]. YFAS and other similar scales record addiction to certain types of food that an individual generally consumes. However, the practice of consuming a specific type of food during or immediately after smoking has seldom been recorded, and the present paper focuses on the development of a unique questionnaire that measures the "secondary food habit" usage among smokers.

Questionnaire constructs were framed based on feedback from preliminary group discussions, and the intended meaning and breadth of each construct were extensively reviewed in the literature, as suggested by Carpenter [16]. Interconnected questions and those with a common context were grouped under a single domain. Likewise, a five-domain construct was generated, with one or more items under each domain. The use of multiple items is intended to measure an underlying construct and to isolate and minimize item-specific measurement error, leading to more accurate research findings [17].

Refinement of the item pool was conducted through focus group discussions and pretesting. Questions that carried similar meaning, were repetitive, and lacked clarity from the participant’s perspective were either removed or modified. The items were refined multiple times with support from expert members, and the finalized item pool was piloted with a minimum number of participants. Weaker items that were retained during the pretesting period were subsequently removed based on responses from the pilot study. Systematic refinement of the item pool with a detailed explanation of removed or modified items is listed in Table 1. The item pool was validated for face and content validity, and the consistency among the questions was also assessed statistically using a reliability assessment. The entire process of this questionnaire validation was carried out by adopting the standard methodologies given in the literature [18-20]. In addition to the items related to secondary food habits, sociodemographic details and details of smoking were also recorded as part of the study.

The questionnaire is unique in that it specifically captures data on the food items used during or immediately after smoking. Previous questionnaires in the literature assessing food intake patterns record consumption throughout the day. In contrast, the present study focuses on the association between the primary habit (smoking) and the secondary habit (intake of food substances). Participants were recruited from diverse settings, as food preferences, their availability, and choices may vary across individuals with different educational levels and occupational profiles. In addition to recording the prevalence, the questionnaire is also targeted at the type of secondary food substances used by smokers, possible reasons for them to choose such food items, and the possible dependency or addiction that the smokers develop toward the food items.

The present study has certain limitations that should be considered while interpreting the findings. Although the questionnaire addresses the gap in understanding food intake patterns associated with smoking, no specific scales were developed in this study to quantify the extent of such practices or to measure the levels of craving, addiction, and dependency. The choice of food substances that smokers prefer during or immediately after smoking varies regionally, as does the reason for such practices. Cross-cultural adaptation and translation should be considered if the questionnaire is intended to record the data from different localities within or outside the country [21]. The study included a higher proportion of male participants than female participants. This gender imbalance may be attributed to the social stigma associated with smoking among women in the Indian cultural context, which could have limited female participation and may affect the generalizability of the findings across both sexes. Furthermore, the sample size was adequate for preliminary validation; however, larger multicentric studies involving diverse populations would strengthen the psychometric properties and external validity of the instrument.

## Conclusions

The choice of food that an individual with a smoking habit prefers to consume immediately or during the smoking episode is related to several physiological, neurochemical, and psychological factors. The present study highlights that a substantial proportion of smokers engage in such “secondary food habits,” a relatively unexplored behavioral pattern. The development of a questionnaire, as in this study, provides a novel and systematic tool for assessing the prevalence, types, and patterns of these habits, along with associated behavioral tendencies. Further research employing this tool across diverse populations and settings may help to identify additional associations and relationships between smoking and secondary food habits, thereby expanding the understanding of this behavior.
